# Toluidine blue versus frozen section for assessment of mucosal tumor margins in oral squamous cell carcinoma

**DOI:** 10.1186/s12885-020-07644-0

**Published:** 2020-11-25

**Authors:** Hana’a Hezam Algadi, Amany Abd-Elhameed Abou-Bakr, Omer Mohammed Jamali, Louloua Mohamed Fathy

**Affiliations:** 1 Department of Oral and Maxillofacial Pathology, Faculty of Dentistry, Cairo University, Cairo, Egypt; 2 Department of Oral and Maxillofacial Pathology, Faculty of Dentistry, Hodeidah University, Hodeidah, Yemen; 3 Department of Pathology, National Cancer Institute, Cairo University, Cairo, Egypt; 4 Department of Oral and Maxillofacial Surgery, Faculty of Dentistry, Cairo University, Cairo, Egypt; 5 Department of Oral and Maxillofacial Surgery, Faculty of Dentistry, Hodeidah University, Hodeidah, Yemen

**Keywords:** Toluidine blue, Frozen section, Tumor margins, Surgical margins, Oral Cancer, Oral squamous cell carcinoma

## Abstract

**Background:**

When the resected specimen is sent for intraoperative margin assessment, all margins are grossly checked, and selected margins undergo a frozen section (FS) examination. Therefore, there is a possibility of sampling error. This study evaluated the effectiveness of using toluidine blue (TB) as an intraoperative triage screening tool to detect positive mucosal margins of the resected specimens of oral squamous cell carcinoma (OSCC) and serve as a guide for FS sampling.

**Methods:**

Surgical samples of 30 consecutive patients with biopsy-proven OSCC were included in the study. A total of 140 mucosal margins were analyzed intraoperatively by TB and FS, the results were compared with the final histopathology.

**Results:**

Of the 140 examined mucosal tumor margins, 14 stained positives with TB, six were true-positives, eight were false-positives, and there were no false-negatives, as confirmed by final histopathology of the same margins. The diagnostic performance measures were sensitivity 100.0%; specificity 94.0%; positive predictive value (PPV) 42.9%; negative predictive value (NPV) 100.0%; and accuracy 94.3% (95% CI: 89.0–97.5%). For FS, there were three true-positives, three false-negatives, and no false-positives. The diagnostic performance measures were sensitivity 50.0%; specificity 100.0%; PPV 100.0%; NPV 97.8%; and accuracy 97.9% (95% CI: 93.9–99.6%).

**Conclusion:**

TB is less specific but more sensitive than FS for detecting positive mucosal margins of resected OSCC. Screening the tumor mucosal margins with TB before FS sampling may help identify more tumor-bearing margins.

**Trial registration:**

This trial was registered at ClinicalTrials.gov. Registration number: NCT03554967. Registration date: June 13, 2018.

## Background

Oral cancer is the eleventh most common malignancy in the world [1]. Oral squamous cell carcinoma (OSCC) is the most common oral cavity malignancy and represents more than 90.0% of oral cancers [2]. The main treatment modality for managing OSCC is surgical resection [3]. A critical issue of surgical oncology is achieving complete removal of a tumor at the primary site with a negative margin. Failure to identify residual neoplastic tissue results in positive surgical margins correlated with local recurrence and poor patient outcomes [4, 5].

Several diagnostic methods have been used for intraoperative identification of tumor-involved margins, including visualization and palpation of the resected margins, touch imprint cytology, microendoscope, fluorescent techniques, Raman spectroscopy, narrow-band imaging, and optical coherence tomography [6–8]. These methods have their challenges and limitations in terms of appropriate performance, practicality, and cost-effectiveness.

A frozen section (FS) is still used in most oncological centers as the standard-of-care means for intraoperative margin assessment. Although it is a reliable method for identifying residual tumor in the sampled tissue, only selected margins-guided by gross examination-are analyzed. Therefore, there is a possibility of sampling error.

Toluidine blue (TB) is a metachromatic stain that is easily available, economical, and has a high affinity for DNA and RNA. It rapidly stains malignant and premalignant cells, but not normal mucosa. Several studies have demonstrated the ability of TB to detect oral cavity premalignant lesions and OSCC [9–12]. However, its use as a screening tool for tumor-involved margins after surgical excision of OSCC has not been extensively studied.

In the literature, only three studies addressed TB intraoperatively. In one study, TB was used intraorally before resection to determine the extent of the lesions [13]. In the other two studies, TB was used intraorally to evaluate the tumor bed margins after excision of primary squamous cell carcinomas (SCC) [14, 15]; Portugal et al. [15] studied the role of TB in assessing margin status after resection of SCC of the upper aerodigestive tract (UADT). The authors applied TB directly to the remaining unresected mucosa. They reported a 100.0% sensitivity in the detection of tumor-involved margins with few false-positives. Junaid et al. [14] evaluated TB staining of tumor bed margins after excision of primary OSCC. Their results indicated a 100.0% sensitivity for detecting positive margins and a specificity of 97.0%.

There are drawbacks to tumor-bed driven margin assessment. In short, tumor bed biopsies are so small, fragmented, unoriented, and not representative of the actual margin status (derived from the main resection specimen) [16, 17]. Recently, Kain et al. performed a systematic review on the surgical margins in oral cavity SCC. This review provides support for the practice of specimen-driven margin assessment when using FS analysis to improve the utility of the results [18].

Due to the drawbacks of tumor-bed driven margin assessment, we decided to test the use of TB in the assessment of the mucosal margin status of a resected OSCC specimen (specimen-driven margin).

The aim of this study was to evaluate the effectiveness of using TB as an intraoperative triage screening tool to detect positive mucosal margins of the resected specimens of OSCC and serve as a guide for FS sampling.

## Methods

This prospective diagnostic accuracy study was designed according to the STARD checklist and was conducted at the Faculty of Dentistry and National Cancer Institute (NCI), Cairo University, Egypt, from July 2018 to June 2019. The accuracies of TB and FS were compared with the final histopathology of the same margins. Thirty consecutive patients with biopsy-proven OSCC who were undergoing primary excision were included in the study. The patients were recruited regardless of age, sex, ethnicity, and tumor stage or grade. The exclusion criteria were patients with a history of head and neck radiotherapy, those with previous treatment (surgery and radio−/ chemotherapy) for current OSCC because increased fibrosis and scar tissue can lead to mechanical retention of TB, making it difficult to interpret staining results. This study was approved by the Research Ethical Committee at the Faculty of Dentistry, Cairo University (number: 10 6 18). This study operated in compliance with the Helsinki Declaration, and written informed consent was obtained from all patients.

All surgical procedures were performed by an experienced head and neck surgeons at Head and Neck Surgical Oncology Unit, NCI, Egypt. After the surgical excision of the tumor, the surgeon marked the surgical margins by surgical sutures and then immediately sent the resected specimen to the Surgical Pathology Unit.

**Fig. 1:**
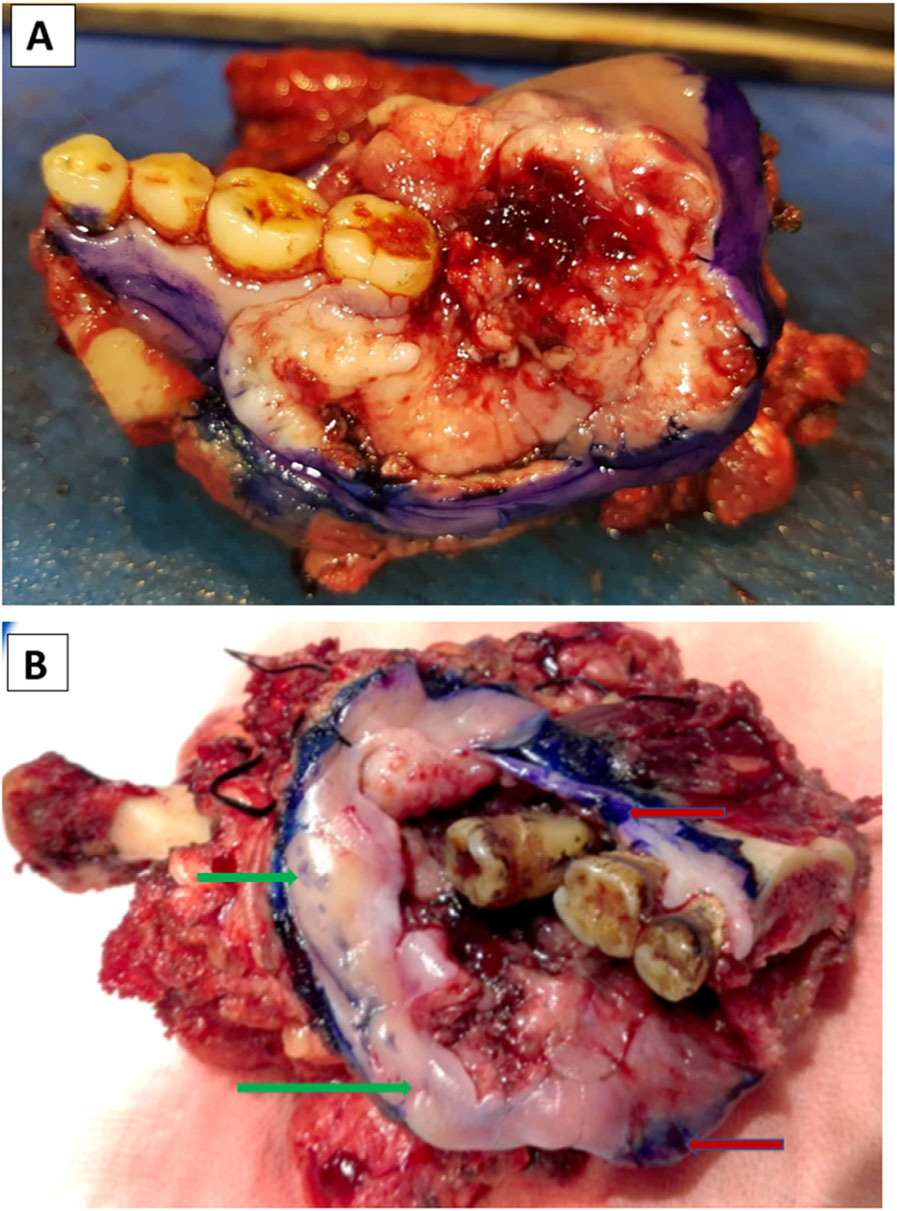
**a** Topical application of 1% TB to the mucosal margins of resected retromolar SCC, **b** another Segmental mandibulectomy specimen (after eliminating the excess of stain); red arrows indicated positive TB staining, green arrow indicated negative TB staining

The mucosal margins were evaluated at two stages:

During the *first stage*, the tissues were assessed intraoperatively (in the laboratory as the operation progressed in the operating room) using:

1. **TB.** The mucosal margins of the resected tumor were stained with TB according to the following protocol:

One percent TB solution staining was conducted as described by Mashberg [9]. Briefly, irrigating the tumor margins was performed first using 1% acetic acid followed by normal saline. The margins were then gently dried with gauze. One percent toluidine blue solution was applied using a cotton swab and left in place for 30 s (Fig. 1a). Thereafter, the tissue was once again irrigated with 1% acetic acid (for eliminating the excess of stain) followed by normal saline. The margins stained in royal blue were labeled as positive, while those stained light blue or unstained were labeled as negative [19] (Fig. 1b).

2) **Hematoxylin and eosin (H&E) stained FS.** A minimum of four samples are shaved off the mucosal margins (specimen margin)—anterior, posterior, medial (or superior), and lateral (or inferior). Additional samples were taken if deemed valuable. Any margin that was positive by TB was included in FS sampling.

The FS interpretation was performed by another histopathologist who was blinded to the result of the TB staining (TB staining does not affect FS interpretation due to the known instability of TB in fixation and dehydration solutions [19]. Even if staining persisted after processing, it would be masked by the H&E stain).

The *second stage (final histopathological)* involved evaluation of FS samples (FS remnants) taken intraoperatively, as described above, following formalin fixing and paraffin embedding and H&E staining. The final histopathological interpretation was performed by another histopathologist who was blinded to the TB and FS results. Resection margins which showed severe dysplasia, carcinoma in situ, or invasive carcinoma, were labeled as positive [20–22]. The severity of the dysplasia was based on the 2017 WHO grading system [23].

### Sample size calculation

A previous paper by Junaid et al. [14] reported that TB staining had a sensitivity and specificity of 100.0 and 97.0%, respectively. Using a 95% confidence interval and a 5% significance level, 30 patients were required. The sample size was calculated by Arifin, W. N. (2017) [24].

### Statistical method

The data were coded and entered using the Statistical Package for the Social Sciences (SPSS) version 25. Data are summarized using means, standard deviations, minimums, and maximums for quantitative data, and frequency (counts), and relative frequency (percentages) for categorical data. Standard diagnostic indices, including sensitivity, specificity, positive predictive value (PPV), negative predictive value (NPV), and diagnostic efficacy were calculated as described by Galen RS [25]. For comparing categorical data, the Chi-square (χ^2^) test was performed. The exact test was used instead when the expected frequency was less than 5 [26]**.** The receiver operator curve (ROC) was constructed and an area under curve analysis was performed to compare the margin status between TB and FS. A *p*-value of less than 0.05 was considered statistically significant.

## Results

Of the 30 patients enrolled in this study, 19 (63.3%) patients were males, and 11 (36.7%) were females. The mean age was 54.4 ± 12.3 years (23–75).

The oral cavity subsites were tongue (*n* = 13; 43.3%), lip (*n* = 4; 13.3%), buccal mucosa (n = 4; 13.3%), retromolar area (n = 4; 13.3%), lower alveolar ridge (*n* = 3; 10.0%), and floor of the mouth (*n* = 2; 6.7%). Regarding the T category of the lesions, according to the seventh edition of the staging of head and neck cancer by the American Joint Committee of Cancer, 3 (10.0%) were T1, 17 (56.7%) were T2, 3 (10.0%) were T3, and 7 (23.3%) were T4.

A total of 140 mucosal margins were analyzed intraoperatively by TB and FS. The results were compared with the final histopathological results of the same margins. Of the 140 examined mucosal surgical margins, there were six (4.3%) positive margins with invasive carcinoma in the final histopathological examination, all these margins were stained positively by TB, and only three (50.0%) of these margins were detected by FS.

For TB, 14/140 (10.0%) margins (14 margins from 11 patients; in three patients, there were two positive margins per patient) were positively stained. However, among these 14 margins, only six (42.9%) were true-positives as confirmed by the final histopathology of the same margins, and eight (57.1%) margins were false-positive (Table 1). There were no false-negatives as confirmed by the final histopathology of the same margins, indicating that TB was able to identify all true-positive margins. Thus, the sensitivity was 100.0% (95% CI: 54.1–100.0%). Eight margins (8/140, 5.7%) were false-positive, as confirmed on the final histopathology, indicating specificity of 94.0% (95% CI: 88.6–97.4%). The diagnostic accuracy of TB was 94.3% (95% CI: 89.0–97.5%) with a PPV of 42.9% (95% CI: 27.7–59.5%) and NPV of 100.0% (Table 2). The sensitivity and NPV remained unchanged in all T categories. In contrast, specificity and PPV were decreased in T3 and T4 tumors (Table 3).

**Table 1:** The results of TB and FS compared with final histopathology results

		Histopathological Diagnoses		
		Positive	Negative	Total
**TB**	**Positive**	6 (TP)	8 (FP)	14 (TP + FP)
	**Negative**	0 (FN)	126 (TN)	126 (FN + TN)
	**Total**	6	134	140 (TP + TN + FP + FN)
**FS**	**Positive**	3 (TP)	0 (FP)	3 (TP + FP)
	**Negative**	3 (FN)	134 (TN)	137 (FN + TN)
	**Total**	6	134	140 (TP + TN + FP + FN)

*Abbreviations: FN* false negative, *FP* false positive, *FS* frozen section, *TB* toluidine blue, *TN* true negative, *TP* true positive

**Table 2:** Accuracy of TB and FS

	Statistic	Value	95% CI
**TB**	Sensitivity	100.0%	54.1 to 100.0%
	Specificity	94.0%	88.6 to 97.4%
	PPV	42.9%	27.7 to 59.5%
	NPV	100.0%	
	Accuracy	94.3%	89.0 to 97.5%
**FS**	Sensitivity	50.0%	11.8 to 88.2%
	Specificity	100.0%	97.3 to 100.0%
	PPV	100.0%	
	NPV	97.8%	95.2 to 99.0%
	Accuracy	97.9%	93.9 to 99.6%

*Abbreviations: CI* Confidence Interval, *FS* frozen section, *NPV* negative predictive value, *PPV* positive predictive value, *TB* toluidine blue

**Table 3:** Sensitivity, specificity, reliability (PPV and NPV) and accuracy of TB and FS for all T categories

		Sensitivity	Specificity	PPV	NPV	Accuracy
**TB**	**overall**	**100.0%**	**94.0%**	**42.9%**	**100.0%**	**94.3%**
	T1	0	100.0%	0	100.0%	100.0%
	T2	100.0%	96.9%	50.0%	100.0%	97.1%
	T3	0	94.4%	0	100.0%	94.4%
	T4	100.0%	86.8%	44.4%	100.0%	88.1%
**FS**	**overall**	**50.00%**	**100.00%**	**100.0%**	**97.8%**	**97.9%**
	T1	0	100.0%	0	100.0%	100.0%
	T2	50.0%	100.0%	100.0%	98.5%	98.5%
	T3	0	100.0%	0	100.0%	100.0%
	T4	50.0%	100.0%	100.0%	95.0%	95.2%

Note: T classification according to the 7th edition of staging of head and neck cancer by the American Joint Committee of cancer

*Abbreviations: FS* frozen sections, *NPV* negative predictive value, *PPV* positive predictive value, TB toluidine blue

**Fig. 2:**
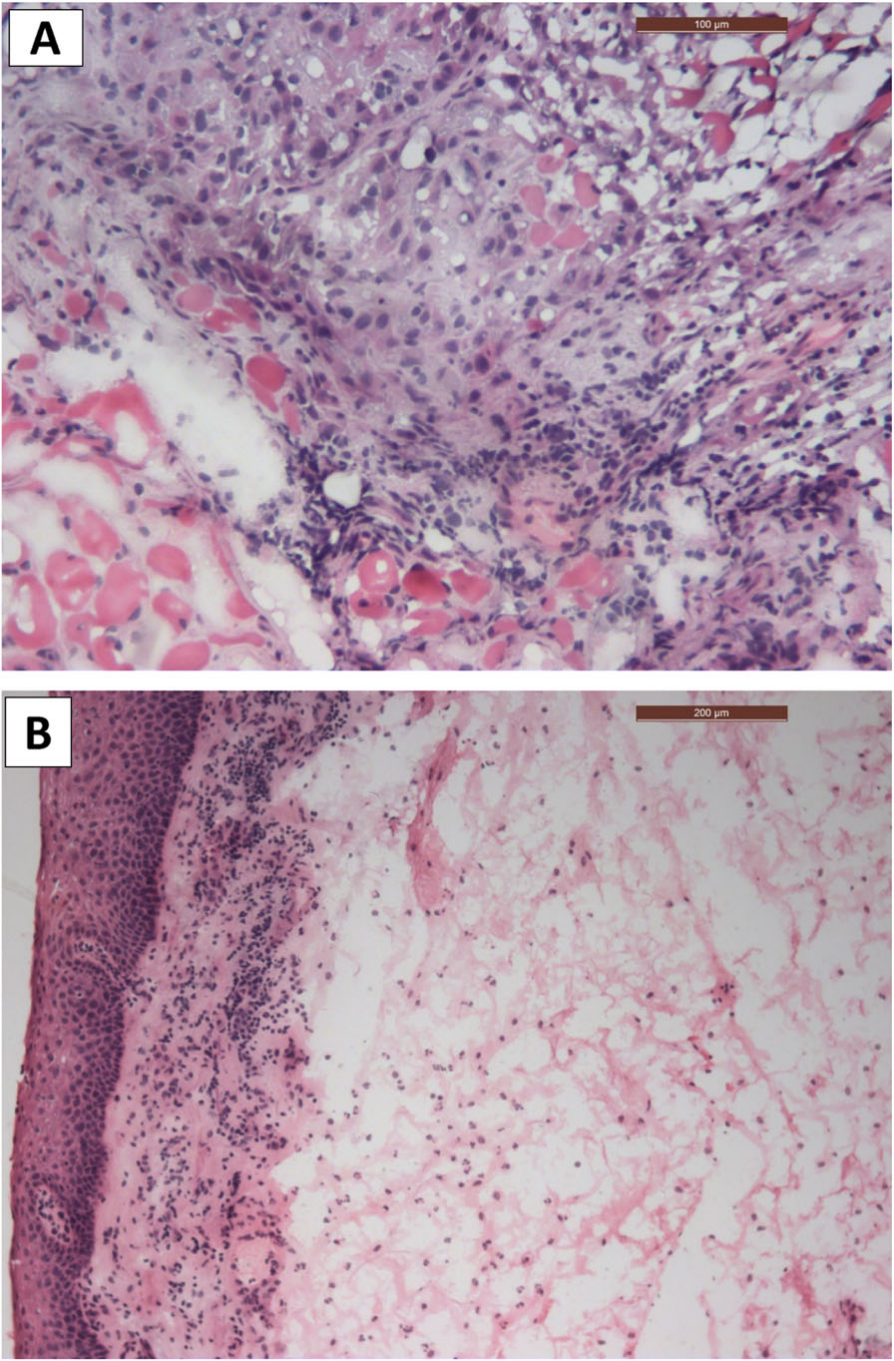
Microscopic photo of FS showing; **a** invasive squamous epithelial cells within connective tissue stroma (positive margin H&E ×100). **b** epithelial surface and connective tissue stroma (negative margin with inflammation, H&E ×200)

**Fig. 3:**
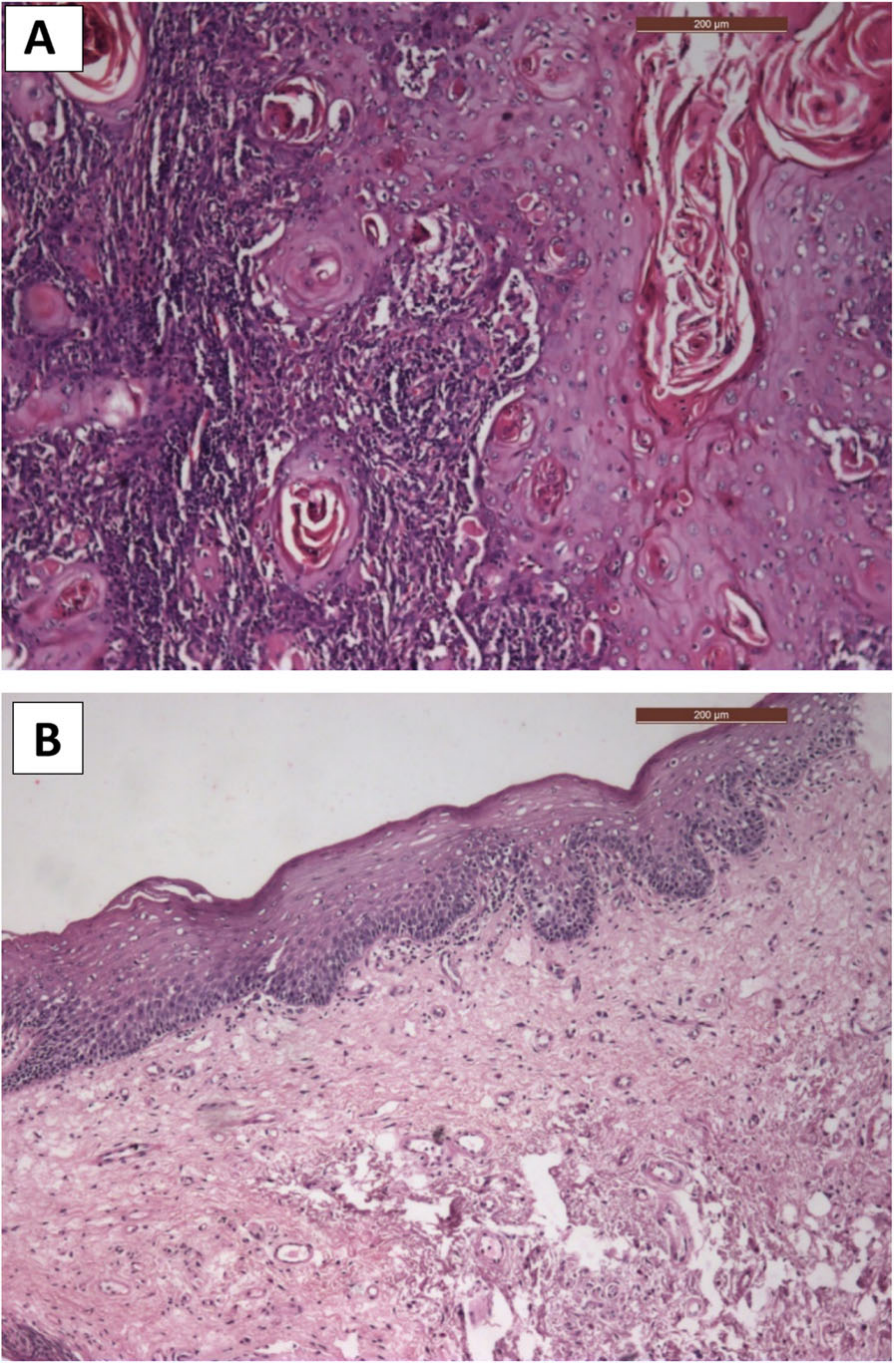
Microscopic photo of final histopathology showing; **a** invasive squamous epithelial cells within connective tissue stroma (positive margin, H&E × 200). **b** normal epithelial surface and connective tissue stroma (negative margin, H&E × 200)

For the FS, there were three true-positives (Fig. 2a), 134 true-negatives (Fig. 2b), three false-negative margins, and no false-positive margins, as confirmed in the final histopathology of the same margins (Fig. 3a, b), (Table 1). The sensitivity of the test was 50.0% (95% CI: 11.8–88.2%) and specificity 100.0% (95% CI: 97.3–100.0%). PPV was 100.0% and NPV was 97.8% (95% CI: 95.2–99.0%). The diagnostic accuracy of FS was 97.9% (95% CI: 93.9–99.6%) (Table 2). The specificity remains unchanged in all T categories, while NPV decreases with T4 (Table 3).

**Fig. 4:**
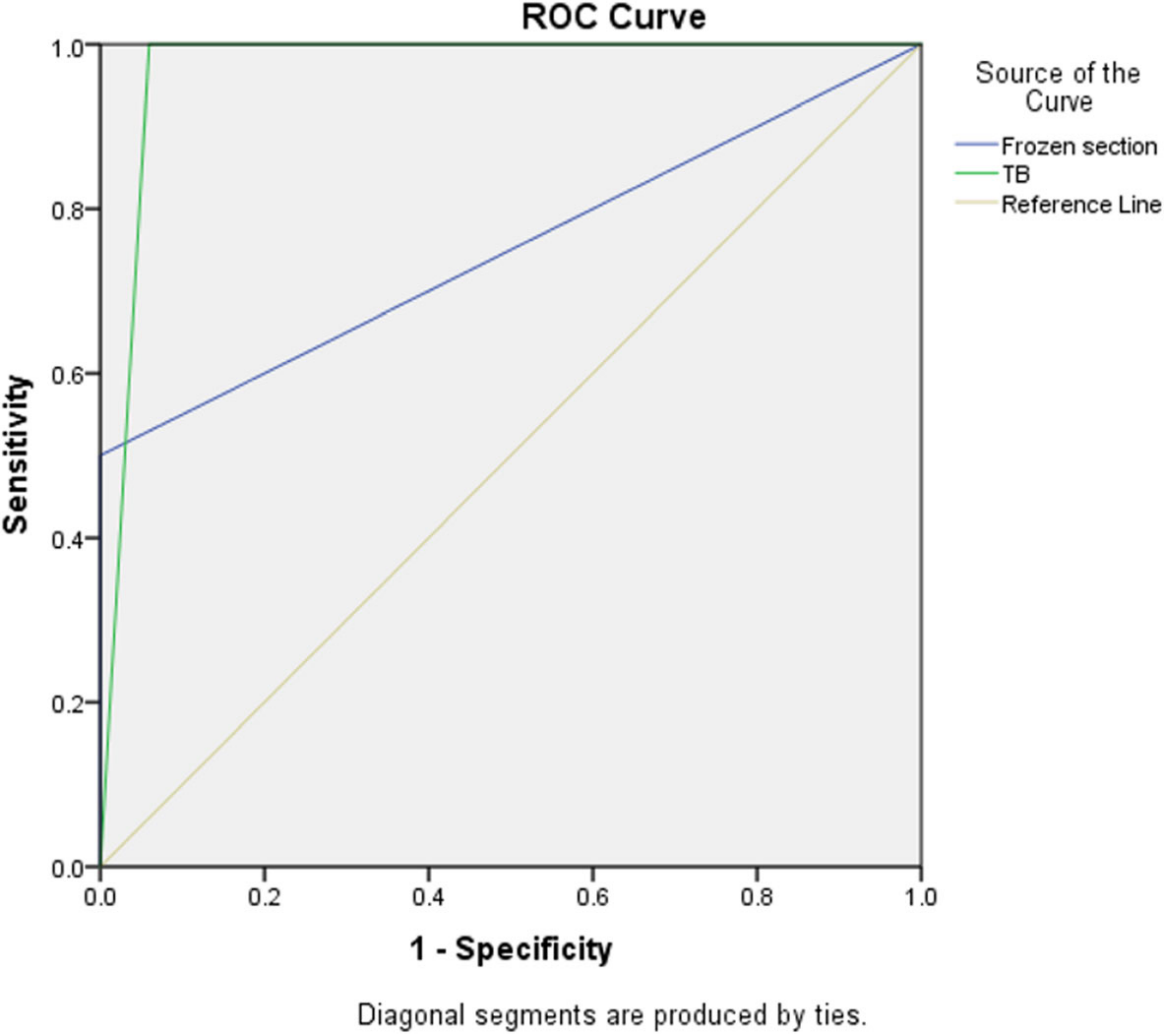
Receiver operator curve showing area under curve for FS =75.0% (95%CI 49.0–100.0%) with sensitivity = 50.0% and specificity = 100.0%, *p* value = 0.039. Area under curve for TB =97% (95%CI 94.2–99.8%) with sensitivity = 100.0% and specificity = 94.0%, p-value< 0.001. Green and blue lines represent FS and TB respectively

Regarding the ROC (Fig. 4) analysis for positive margins, we found that the area under the curve for FS was 75.0% (95% CI 49.0–100.0%) with a sensitivity of 50.0% and a specificity of 100.0% (*p*-value = 0.039). The area under the curve for TB was 97.0% (95%CI 94.2%–99.8%) with a sensitivity of 100.0% and specificity of 94.0% (*p* < 0.001).

## Discussion

TB is a metachromatic and affordable stain with a high affinity for DNA and RNA. High DNA and RNA content in actively growing tissue—such as malignant proliferation and wider intercellular canals compared to normal epithelial cells—is responsible for staining malignant lesions [27]. Since the 1960s, TB has been used in vivo as a screening tool. It has demonstrated its ability to generate malignant and premalignant cell stains, but not normal mucosa [28].

The ability of TB to detect oral and oropharyngeal carcinoma is well documented. The sensitivity and specificity rates for use of TB as a screening tool for oral premalignant and malignant lesions ranged from 77.0–100.0% and 65.5–100.0%, respectively [11, 29–32]. Onofre et al. [11] and Warnakulasuriya et al. [30] reported that TB was 100.0% sensitive for detecting oral carcinoma without false-negative results. Epstein et al. [33] reported that TB was 96.7% sensitive for detecting recurrence or second primary cancers in patients previously treated for UADT malignancies.

TB can more accurately assess the superficial extent of a lesion before its excision, thus adding confidence in tumor excision within safe surgical margins. It can also be used to assess margin status after tumor resection [34]. However, only two studies addressed the use of TB for assessing tumor bed margins after excision of primary SCC [14, 15], and its use in assessing specimen-driven margins has not been studied.

We examined use of TB as a triage-screening tool for detecting positive mucosal margins of the main OSCC specimen (specimen-driven margin), thereby guiding FS sampling. We compared the results of TB to FS (standard of care) and final histopathology (gold standard) of the same margins.

TB had a sensitivity of 100.0% and a specificity of 94.0% with a NPV of 100.0% and a PPV of 42.9%. Our results are in general agreement with previous studies [14, 15] regarding the ability of TB to identify all positive mucosal tumor margins with no false-negatives and a slightly lower specificity.

In this study, 14 margins were stained positively with TB. Six were true-positive, as confirmed by the final histopathological examination of the same margins. The eight false-positive margins slightly reduced the TB specificity and our results were lower than the findings of Junaid et al. **[**14] who reported a TB specificity of 97.0%. Six of these eight false-positive margins were attributed to surgical traumatic handling of the mucosa. This led to the exposure of submucosal connective tissue which facilitated TB uptake by the nuclear material of the injured exposed cells. In the remaining two false-positive margins, TB staining was caused by extensive inflammatory cell foci. These were observed during the FS examination and in the final histopathological examination of the same margins. At the site of inflammation, there is an increase in both cellular activity and mechanical retention, which facilitating TB retention [35].

Although our PPV was high (42.9%) compared to the 27.2% in the study by Junaid et al. [14], it is still sub-optimal. PPV is inversely proportional to the number of false-positive margins, which were most often related to the traumatic handling of the mucosa during the resection. This indicates that careful tissue handling during excision may reduce false-positive results and increases the PPV. However, TB staining had NPV of 100.0%, indicating that it identified all positive mucosal tumor margins.

When considering the T category of the tumor, the false-positive margins were two in T2, one in T3, and five in T4. Similar to Junaid’s results [14], most of the false-positives were in the T4. All five false-positive T4 stains were attributed to mucosal trauma sustained during the intraoperative handling of large-size tumors. This exposed the submucosal connective tissue and facilitated retention of the TB.

In his study, Portugal et al. [15] were reported that TB staining of the surrounding unresected mucosa identified three cases of a second primary tumor that were not previously identified. We cannot comment on identifying second primary tumors as this was not detected in our study.

FS identified three of six positive margins, indicating a sensitivity of 50.0%. These results are close to those of Mair et al. [36] who reported that FS had a sensitivity of 45.4%. Our findings were inconsistent with some previous studies that reported FS sensitivity of 72.0–100.0% [14, 37–39]. The three false negatives that reduced the sensitivity of FS in our study were mainly attributed to interpretation errors rather than sampling errors (because all margins that were positive by TB were included in the FS sampling). The interpretation errors occurred as a result of inherent artifacts caused by ice crystal formation, or by distortion of the tissue architecture during the freezing process. These artifacts included darkened nuclei and nuclei angulation changes. These factors make it more difficult to recognize malignant cells and thus complicates the interpretation of the FS.

All positive FS margins were also positive in the final histopathological examination of the same margins with no false-positives, indicating a 100.0% specificity. Our results were similar to those of Junaid et al. [14] who found that FS had a specificity of 100.0%. When comparing margin discrepancies based on the T category, we found two false-negative margins in T4 and one false-negative in T2. Notably, our study had a small sample size and it was not possible to determine if the intraoperative false-negative results of the FS specimens and T category depend on each other. This would require comparing the FS results within only one T category.

A triage test is used to rule out disease (not to rule in disease), and therefore needs very high sensitivity [40]. The result of this study showed that TB is highly sensitive and can be used as a triage-screening tool for positive OSCC mucosal margins. Moreover, TB can reduce the time and cost of FS. The number of FS biopsies can be limited to the positive stained mucosal margins plus a deep (non-mucosal) margin.

Although TB has a high sensitivity for positive OSCC mucosal margins, this test is not without limitations. TB is less-specific than FS. Also, resected tumor margin scars that result from previous chemotherapy, radiotherapy, or surgery may cause TB retention, potentially producing false-positive results. Also, TB can only be used to assess mucosal margins and has no diagnostic value for deep (non-mucosal) margins of resected specimens since it is readily absorbed by connective tissue.

## Conclusion

TB is less-specific but more sensitive than FS for detecting positive mucosal margins of resected OSCC. Screening the tumor mucosal margins with TB before FS sampling may help identify more tumor-bearing margins.

## Data Availability

The datasets used and/or analyzed during the current study are available from the corresponding author on reasonable request.

## Abbreviations

FS: Frozen section
H&E: Haematoxylin and eosin
NPV: Negative predictive value
NCI: National Cancer Institute
OSCC: Oral squamous cell carcinoma
PPV: Positive predictive value
ROC: Receiver operator curve
SCC: Squamous Cell Carcinoma
TB: Toluidine blue
T: tumor size
